# Supplementary material for: Desalination Characteristics of Cellulose Acetate FO Membrane Incorporated with ZIF-8 Nanoparticles
Source: Membranes (Basel). 2022 Jan 21;12(2):122. doi: 10.3390/membranes12020122 (PMC8877917; doi:10.3390/membranes12020122)
Supplement: Supplementary file 1 [file membranes-12-00122-s001.zip › membranes-1555978-supplementary.pdf]

Supplementary Materials:

Table S1 Parameters of membrane preparation.

CA	14 wt%
PEG-400	8.0 wt%
1,4-dioxane	6.0 wt%
ZIF-8	0.2~1.0 wt%
Mixing temperature	40~80 °C
Coagulation bath temperature	25~45 °C
Heat treatment temperature	40~80 °C

Table S2 Parameters of membrane testing.

Temperature	At room temperature
Extraction solution	1M NaCl
Raw material	Deionized water
Peristaltic pump rotating speed	100 rpm
